# Supplementary material for: Early urine proteome changes in an implanted bone cancer rat model
Source: Bone Rep. 2019 Dec 13;12:100238. doi: 10.1016/j.bonr.2019.100238 (PMC6921149; doi:10.1016/j.bonr.2019.100238)
Supplement: Supplementary file 1 — Supplementary tables [file mmc1.docx]

**Table S1:** **Biological processes of differential proteins.**

| **Category** | **Term** | **Count** | **%** | **P-Value** |
| --- | --- | --- | --- | --- |
| **D3** |  |  |  |  |
| GOTERM_BP_DIRECT | response to organic substance | 4 | 16 | 0.0011 |
| GOTERM_BP_DIRECT | acute-phase response | 3 | 12 | 0.0012 |
| GOTERM_BP_DIRECT | cellular response to lipopolysaccharide | 4 | 16 | 0.0015 |
| GOTERM_BP_DIRECT | oxidation-reduction process | 6 | 24 | 0.0016 |
| GOTERM_BP_DIRECT | adaptive immune response | 3 | 12 | 0.0042 |
| GOTERM_BP_DIRECT | innate immune response | 4 | 16 | 0.0052 |
| GOTERM_BP_DIRECT | response to drug | 5 | 20 | 0.0053 |
| GOTERM_BP_DIRECT | myeloid cell homeostasis | 2 | 8 | 0.015 |
| GOTERM_BP_DIRECT | response to oxidative stress | 3 | 12 | 0.017 |
| GOTERM_BP_DIRECT | positive regulation of receptor-mediated endocytosis | 2 | 8 | 0.026 |
| GOTERM_BP_DIRECT | muscle cell cellular homeostasis | 2 | 8 | 0.027 |
| GOTERM_BP_DIRECT | cellular response to interleukin-6 | 2 | 8 | 0.038 |
| GOTERM_BP_DIRECT | iron ion homeostasis | 2 | 8 | 0.043 |
| GOTERM_BP_DIRECT | retina homeostasis | 2 | 8 | 0.047 |
| GOTERM_BP_DIRECT | cell adhesion | 3 | 12 | 0.049 |
|  |  |  |  |  |
| **D5** |  |  |  |  |
| GOTERM_BP_DIRECT | response to copper ion | 3 | 23.1 | 0.00017 |
| GOTERM_BP_DIRECT | response to carbon monoxide | 2 | 15.4 | 0.0041 |
| GOTERM_BP_DIRECT | cellular response to lipopolysaccharide | 3 | 23.1 | 0.0056 |
| GOTERM_BP_DIRECT | cell adhesion | 3 | 23.1 | 0.013 |
| GOTERM_BP_DIRECT | cell aging | 2 | 15.4 | 0.022 |
| GOTERM_BP_DIRECT | retina homeostasis | 2 | 15.4 | 0.024 |
| GOTERM_BP_DIRECT | positive regulation of nitric oxide biosynthetic process | 2 | 15.4 | 0.03 |
| GOTERM_BP_DIRECT | response to amphetamine | 2 | 15.4 | 0.031 |
| GOTERM_BP_DIRECT | embryo implantation | 2 | 15.4 | 0.036 |
| GOTERM_BP_DIRECT | ovarian follicle development | 2 | 15.4 | 0.041 |
| GOTERM_BP_DIRECT | response to drug | 3 | 23.1 | 0.049 |
|  |  |  |  |  |
| **D7** |  |  |  |  |
| GOTERM_BP_DIRECT | cellular response to lipopolysaccharide | 4 | 19 | 0.00086 |
| GOTERM_BP_DIRECT | response to cadmium ion | 3 | 14.3 | 0.0012 |
| GOTERM_BP_DIRECT | cell adhesion | 4 | 19 | 0.0031 |
| GOTERM_BP_DIRECT | Aging | 4 | 19 | 0.0052 |
| GOTERM_BP_DIRECT | myeloid cell homeostasis | 2 | 9.5 | 0.012 |
| GOTERM_BP_DIRECT | positive regulation of blood coagulation | 2 | 9.5 | 0.016 |
| GOTERM_BP_DIRECT | Fibrinolysis | 2 | 9.5 | 0.017 |
| GOTERM_BP_DIRECT | response to ethanol | 3 | 14.3 | 0.02 |
| GOTERM_BP_DIRECT | cellular response to cadmium ion | 2 | 9.5 | 0.021 |
| GOTERM_BP_DIRECT | cellular response to interleukin-6 | 2 | 9.5 | 0.031 |
| GOTERM_BP_DIRECT | response to copper ion | 2 | 9.5 | 0.033 |
| GOTERM_BP_DIRECT | innate immune response | 3 | 14.3 | 0.035 |
| GOTERM_BP_DIRECT | iron ion homeostasis | 2 | 9.5 | 0.036 |
| GOTERM_BP_DIRECT | cell aging | 2 | 9.5 | 0.036 |
| GOTERM_BP_DIRECT | retina homeostasis | 2 | 9.5 | 0.039 |
| GOTERM_BP_DIRECT | leukocyte migration | 2 | 9.5 | 0.039 |
| GOTERM_BP_DIRECT | acute-phase response | 2 | 9.5 | 0.042 |
| GOTERM_BP_DIRECT | cellular iron ion homeostasis | 2 | 9.5 | 0.046 |
|  |  |  |  |  |
| **D13** |  |  |  |  |
| GOTERM_BP_DIRECT | positive regulation of gene expression | 5 | 19.2 | 0.0018 |
| GOTERM_BP_DIRECT | response to estradiol | 4 | 15.4 | 0.0028 |
| GOTERM_BP_DIRECT | positive regulation of keratinocyte apoptotic process | 2 | 7.7 | 0.0043 |
| GOTERM_BP_DIRECT | response to drug | 5 | 19.2 | 0.0062 |
| GOTERM_BP_DIRECT | wound healing | 3 | 11.5 | 0.014 |
| GOTERM_BP_DIRECT | apoptotic process | 4 | 15.4 | 0.015 |
| GOTERM_BP_DIRECT | response to glucocorticoid | 3 | 11.5 | 0.015 |
| GOTERM_BP_DIRECT | phospholipid efflux | 2 | 7.7 | 0.02 |
| GOTERM_BP_DIRECT | response to ethanol | 3 | 11.5 | 0.031 |
| GOTERM_BP_DIRECT | cholesterol efflux | 2 | 7.7 | 0.038 |
| GOTERM_BP_DIRECT | lipoprotein metabolic process | 2 | 7.7 | 0.038 |
| GOTERM_BP_DIRECT | cellular response to platelet-derived growth factor stimulus | 2 | 7.7 | 0.041 |
| GOTERM_BP_DIRECT | limb development | 2 | 7.7 | 0.049 |
|  |  |  |  |  |

**Table S2: Cellular components of differential proteins**

| **Category** | **Term** | **Count** | **%** | **P-Value** |
| --- | --- | --- | --- | --- |
| **D3** |  |  |  |  |
| GOTERM_CC_DIRECT | extracellular space | 14 | 56 | 1.3E-09 |
| GOTERM_CC_DIRECT | extracellular exosome | 15 | 60 | 6.8E-07 |
| GOTERM_CC_DIRECT | Lysosome | 4 | 16 | 0.0037 |
| GOTERM_CC_DIRECT | neuronal cell body | 5 | 20 | 0.0048 |
| GOTERM_CC_DIRECT | external side of plasma membrane | 4 | 16 | 0.0048 |
| GOTERM_CC_DIRECT | blood microparticle | 3 | 12 | 0.01 |
| GOTERM_CC_DIRECT | membrane raft | 3 | 12 | 0.046 |
|  |  |  |  |  |
| **D5** |  |  |  |  |
| GOTERM_CC_DIRECT | extracellular exosome | 9 | 69.2 | 0.00005 |
| GOTERM_CC_DIRECT | extracellular space | 5 | 38.5 | 0.0079 |
| GOTERM_CC_DIRECT | external side of plasma membrane | 3 | 23.1 | 0.012 |
| GOTERM_CC_DIRECT | MHC class I protein complex | 2 | 15.4 | 0.015 |
| GOTERM_CC_DIRECT | mitochondrial intermembrane space | 2 | 15.4 | 0.045 |
|  |  |  |  |  |
| **D7** |  |  |  |  |
| GOTERM_CC_DIRECT | extracellular space | 12 | 57.1 | 2.1E-08 |
| GOTERM_CC_DIRECT | extracellular exosome | 13 | 61.9 | 2.9E-06 |
| GOTERM_CC_DIRECT | external side of plasma membrane | 4 | 19 | 0.0028 |
| GOTERM_CC_DIRECT | blood microparticle | 3 | 14.3 | 0.0071 |
| GOTERM_CC_DIRECT | extracellular matrix | 3 | 14.3 | 0.03 |
| GOTERM_CC_DIRECT | extracellular region | 4 | 19 | 0.04 |
|  |  |  |  |  |
| **D13** |  |  |  |  |
| GOTERM_CC_DIRECT | extracellular exosome | 23 | 88.5 | 3.5E-16 |
| GOTERM_CC_DIRECT | extracellular space | 13 | 50 | 3.4E-08 |
| GOTERM_CC_DIRECT | blood microparticle | 5 | 19.2 | 0.000018 |
| GOTERM_CC_DIRECT | extracellular region | 8 | 30.8 | 0.000032 |
| GOTERM_CC_DIRECT | Lysosome | 4 | 15.4 | 0.0042 |
| GOTERM_CC_DIRECT | Cytosol | 7 | 26.9 | 0.017 |
| GOTERM_CC_DIRECT | high-density lipoprotein particle | 2 | 7.7 | 0.023 |
| GOTERM_CC_DIRECT | very-low-density lipoprotein particle | 2 | 7.7 | 0.023 |
| GOTERM_CC_DIRECT | myelin sheath | 3 | 11.5 | 0.027 |
| GOTERM_CC_DIRECT | cell surface | 4 | 15.4 | 0.048 |
|  |  |  |  |  |

**Table S3: Differential protein statistics at various time points in the rat model of tumor bone metastasis and their association with related diseases.**

| **UniProt ID** | **Protein name** | **Human homologous protein ID** | **Fold change(E/C)** | | | | **PubMed** | | | | |
| --- | --- | --- | --- | --- | --- | --- | --- | --- | --- | --- | --- |
|  |  |  | **D3/C** | **D5/C** | **D7/C** | **D13/C** | **Tumor Bone metastasis** | **cancer** | | **Orthopedic disease** | |
| **P16067** | **Atrial natriuretic peptide receptor 2** | **P20594** | **63.30** |  |  |  |  |  | |  | |
| **O70513** | **Galectin-3-binding protein** | **Q08380** | **17.67** | **13.13** | **4.64** |  |  | **PMID: 17608509** | | **PMID: 14558084** | |
| **Q99MH3** | **Hepcidin** | **P81172** | **11.91** |  | **12.61** |  |  | **PMID: 29434930;** | | **PMID: 24014495** | |
| **P08721** | **Osteopontin** | **P10451** | **10.30** |  |  |  | **PMID: 25220832** | **PMID: 28860821** | | **PMID: 27357308** | |
| **Q9Z0J6** | **Growth/differentiation factor 15** | **Q99988** | **8.89** |  |  |  | **PMID: 22370725** | **PMID:22370725;**  **PMID:24920244;**  **PMID:19924834** | | | |
| **Q07523** | **Hydroxyacid oxidase 2** | **Q9NYQ3** | **7.90** |  |  |  |  |  | | |  |
| **O70215** | **NKG2-D type II integral membrane protein** | **P26718** | **6.84** | **6.70** | **7.20** |  |  |  | | |  |
| **P55051** | **Fatty acid-binding protein, brain** | **O15540** | **5.79** |  |  |  |  | **PMID:21683222;**  **PMID:22562177;**  **PMID:24274717** | | | |
| **Q811M5** | **Complement component C6** | **P13671** | **5.23** |  |  |  |  | **PMID:11378347** | | | |
| **P02764** | **Alpha-1-acid glycoprotein** | **P02763** | **5.06** |  |  |  |  | **PMID:27186407;**  **PMID: 26586387** | | | **PMID: 26915672** |
| **P97615** | **Thioredoxin, mitochondrial** | **Q99757** | **4.60** |  |  |  |  | **PMID: 25109980** | | | **PMID: 11841836** |
| **P07151** | **Beta-2-microglobulin** | **P61769** | **4.49** | **3.68** | **12.44** |  | **PMID: 21427356;**  **PMID: 16982753** | **PMID: 3551779 PMID: 28730765** | | | **PMID: 18795399** |
| **P27590** | **Uromodulin** | **P07911** | **4.47** |  |  |  |  |  | | |  |
| **Q566E6** | **CMRF35-like molecule 1** | **Q8TDQ1** | **4.40** |  |  |  |  |  | | |  |
| **Q80WY6** | **Tumor necrosis factor receptor superfamily member 1B** | **P20333** | **3.95** |  |  |  | **PMID: 23493346** | **PMID: 28789455;**  **PMID: 28123565** | | | **PMID: 26071216** |
| **P07632** | **Superoxide dismutase [Cu-Zn]** | **P00441** | **3.52** | **1.82** | **21.27** |  |  | **PMID: 23558240** | | | |
| **Q02765** | **Cathepsin S** | **P25774** | **3.47** |  |  |  |  | **PMID: 27058412; PMID: 25872877** | | | **PMID: 26060322; PMID: 26941186** |
| **P23764** | **Glutathione peroxidase 3** | **P22352** | **3.05** |  |  |  |  |  | | |  |
| **Q562C9** | **1,2-dihydroxy-3-keto-5-methylthiopentene dioxygenase** | **Q9BV57** | **2.72** |  |  |  |  |  | | |  |
| **Q9EPF2** | **Cell surface glycoprotein MUC18** | **P43121** | **2.70** |  | **3.26** |  | **PMID: 14695161;** | **PMID: 26617818;**  **PMID: 25510693;**  **PMID: 10211875** | | | **PMID: 10211875** |
| **P06399** | **Fibrinogen alpha chain** | **P02671** | **2.68** |  |  |  |  | **PMID: 28481733;**  **PMID: 28376075** | | | |
| **Q01205** | **Dihydrolipoyllysine-residue succinyltransferase component of 2-oxoglutarate dehydrogenase complex, mitochondrial** | **P36957** | **2.50** |  |  |  |  |  | | |  |
| **Q6P7A9** | **Lysosomal alpha-glucosidase** | **P10253** | **2.05** |  |  |  |  |  | | |  |
| **P15978** | **Class I histocompatibility antigen, Non-RT1.A alpha-1 chain** | **P16189** |  | **10.05** |  |  |  |  | | |  |
| **P62898** | **Cytochrome c, somatic** | **P99999** |  | **3.86** |  |  |  | **PMID: 21034352;** | | |  |
| **Q5BK81** | **Prostaglandin reductase 2** | **Q8N8N7** |  | **3.48** |  |  |  |  | | |  |
| **Q00238** | **Intercellular adhesion molecule 1** | **P05362** |  | **2.32** | **4.19** |  | **PMID: 26503469** | **PMID: 21590495;**  **PMID: 20495107;** | | | **PMID: 29556418;** |
| **P23785** | **Granulins** | **P28799** |  | **2.27** | **2.66** |  |  | **PMID: 26000098;**  **PMID: 18455118** | | | |
| **Q6DGG1** | **Protein ABHD14B** | **Q96IU4** |  | **2.17** | **14.65** |  |  |  | | |  |
| **P02803** | **Metallothionein-1** | **P04731** |  |  | **142.23** |  |  | **PMID: 27184800; PMID: 23662831** | | | **PMID: 30055006** |
| **P08649** | **Complement C4** | **P0C0L4** |  |  | **9.96** |  |  | **PMID: 27620295** | | | **PMID: 27716448;**  **PMID: 27914370** |
| **O70540** | **Mucosal addressin cell adhesion molecule 1** | **Q13477** |  |  | **8.43** |  |  |  | | | **PMID: 15283850** |
| **Q9WUC4** | **Copper transport protein ATOX1** | **O00244** |  |  | **4.81** |  |  |  | | |  |
| **P85971** | **6-phosphogluconolactonase** | **O95336** |  |  | **4.33** |  | **PMID: 28260828** | | | |  |
| **P53369** | **7,8-dihydro-8-oxoguanine triphosphatase** | **P36639** |  |  | **4.06** |  |  | |  | |  |
| **P18292** | **Prothrombin** | **P00734** |  |  | **3.48** |  |  | |  | |  |
| **P29315** | **Ribonuclease inhibitor** | **P13489** |  |  | **2.88** |  |  | |  | |  |
| **P62815** | **V-type proton ATPase subunit B, brain isoform** | **P21281** |  |  |  | **13.52** |  | |  | |  |
| **P0C6C0** | **A-kinase anchor protein SPHKAP** | **Q2M3C7** |  |  |  | **13.20** |  | |  | |  |
| **P20767** | **Ig lambda-2 chain C region** | **P0DOY2** |  |  |  | **11.23** |  | |  | |  |
| **P19629** | **L-lactate dehydrogenase C chain** | **P07864** |  |  |  | **8.52** |  | |  | |  |
| **Q9Z2L0** | **Voltage-dependent anion-selective channel protein 1** | **P21796** |  |  |  | **8.07** |  | |  | |  |
| **P21674** | **Follistatin** | **P19883** |  |  |  | **6.58** | **PMID: 20623366** | | | |  |
| **P11348** | **Dihydropteridine reductase** | **P09417** |  |  |  | **6.13** |  | |  | |  |
| **P45479** | **Palmitoyl-protein thioesterase 1** | **P50897** | **0.29** |  |  |  |  | |  | |  |
| **Q5U367** | **Procollagen-lysine,2-oxoglutarate 5-dioxygenase 3** | **O60568** | **0.17** | **0.20** |  |  | **PLOD2**  **is associated with bone metastasis** | | **PMID: 29644003;**  **PMID: 29059470;** | |  |
| **P53790** | **Sodium/glucose cotransporter 1** | **P13866** |  | **0.39** | **0.18** |  |  | | **PMID: 23765757;** | | |
| **Q04807** | **Glycosylation-dependent cell adhesion molecule 1** | **Q8IVK1** |  | **0.20** |  |  |  | |  | |  |
| **P04276** | **Vitamin D-binding protein** | **P02774** |  |  | **0.43** | **0.40** |  | |  | | **PMID: 22704802** |
| **D3ZTE0** | **Coagulation factor XII** | **P00748** |  |  | **0.24** |  |  | |  | |  |
| **P98158** | **Low-density lipoprotein receptor-related protein 2** | **P98164** |  |  |  | **0.56** |  | |  | | **PMID: 26147675** |
| **Q4QQW8** | **Putative phospholipase B-like 2** | **Q8NHP8** |  |  |  | **0.48** |  | |  | |  |
| **Q62867** | **Gamma-glutamyl hydrolase** | **Q92820** |  |  |  | **0.45** |  | | **PMID: 28146062;**  **PMID: 23374458** | | |
| **P02651** | **Apolipoprotein A-IV** | **P06727** |  |  |  | **0.45** |  | | **PMID: 29123261;**  **PMID: 27974108** | | **PMID: 28445313** |
| **P20059** | **Hemopexin** | **P02790** |  |  |  | **0.45** |  | |  | |  |
| **P04937** | **Fibronectin** | **P02751** |  |  |  | **0.44** | **PMID: 25623744** | | **PMID: 27250024** | | |
| **P14925** | **Peptidylglycine alpha-amidating monooxygenase** | **P19021** |  |  |  | **0.42** |  | |  | |  |
| **P11232** | **Thioredoxin** | **P10599** |  |  |  | **0.40** |  | |  | |  |
| **P48199** | **C-reactive protein** | **P02741** |  |  |  | **0.40** |  | |  | |  |
| **P19939** | **Apolipoprotein C-I** | **P02654** |  |  |  | **0.39** |  | |  | | **PMID: 23935359** |
| **Q63347** | **26S protease regulatory subunit 7** | **P35998** |  |  |  | **0.38** |  | |  | |  |
| **P35444** | **Cartilage oligomeric matrix protein** | **P49747** |  |  |  | **0.36** |  | |  | |  |
| **Q9EPB1** | **Dipeptidyl peptidase 2** | **Q9UHL4** |  |  |  | **0.36** | **PMID：6089828** | | | |  |
| **P21704** | **Deoxyribonuclease-1** | **P24855** |  |  |  | **0.34** |  | |  | |  |
| **Q68FP1** | **Gelsolin** | **P06396** |  |  |  | **0.34** |  | |  | |  |
| **Q5XIE8** | **Integral membrane protein 2B** | **Q9Y287** |  |  |  | **0.34** |  | |  | |  |
| **Q76HN1** | **Hyaluronidase-1 SV=1** | **Q12794** |  |  |  | **0.32** |  | |  | |  |
| **Q9JLS4** | **Secreted frizzled-related protein 4** | **Q6FHJ7** |  |  |  | **0.32** |  | |  | |  |
| **P80202** | **Activin receptor type-1B** | **P36896** |  |  |  | **0.19** |  | |  | |  |
